# Effect of Inter-Observer Variation on the Association between Contamination Hazards and the Microbiological Quality of Water Sources: A Longitudinal Study

**DOI:** 10.3390/ijerph17249192

**Published:** 2020-12-09

**Authors:** Joseph Okotto-Okotto, Diogo Trajano Gomes da Silva, Emmah Kwoba, Samuel.M Thumbi, Peggy Wanza, Weiyu Yu, Jim A. Wright

**Affiliations:** 1 Victoria Institute for Research on Environment and Development (VIRED) International, P.O. Box 6423-40103, off Nairobi Road, Rabour, Kisumu, Kenya; jokotto@hotmail.com; 2 School of Environment and Technology, University of Brighton, Cockcroft Building, Lewes Road, Brighton BN2 4GJ, UK; d.trajanogomesdasilva2@brighton.ac.uk; 3 International Livestock Research Institute (ILRI), P.O. Box 30709-00100, Naivasha Road, Nairobi, Kenya; emmiesusan38@gmail.com; 4 Centre for Global Health Research, Kenya Medical Research Institute, P.O. Box 1578-40100, Kisumu, Kenya; thumbi.mwangi@wsu.edu (S.T.); peggywanza@gmail.com (P.W.); 5 Institute of Tropical and Infectious Diseases, University of Nairobi, P.O. Box 19676-00202, Nairobi, Kenya; 6 Paul G Allen School for Global Animal Health, Washington State University, P.O. Box 647090, Pullman, WA 99164, USA; 7 School of Geography and Environmental Science, University of Southampton, Building 44, Highfield, Southampton SO17 1BJ, UK; W.Yu@soton.ac.uk

**Keywords:** *Escherichia coli*, enterococcus, water supply, water pollution, cattle, Kenya

## Abstract

Sanitary risk inspection protocols are often used to identify contamination hazards at water sources; however, different observers sometimes struggle to record hazards consistently. This study aimed to assess the effect of inter-observer variation in hazard observations on the strength of relationships between observed hazards and the bacterial contamination of water sources, particularly relationships with animal-related hazards. In a longitudinal study, five surveyors independently recorded hazards at 93 water sources used by 234 households in Siaya County, Kenya, in both wet and dry seasons. One surveyor collected samples from sources for subsequent *Escherichia coli* and intestinal enterococci testing. The relationship between each surveyor’s hazard observations and high bacterial contamination was examined using logistic regression. After controlling for water source type and preceding rainfall; percentage scores for animal-related hazards were significantly related to high contamination with enterococci and *E. coli* for one surveyor (odds ratio 1.02; 95% confidence intervals 1.00–1.03 for both parameters), but not for the remaining four surveyors. The relationship between observed contamination hazards and the microbiological contamination of water sources is sensitive to variation in hazard recording between surveyors. Sanitary risk protocols should be designed to enable robust and consistent observation of hazards.

## 1. Introduction

An estimated 1.8 billion people use faecally contaminated water, with 1.1 billion using drinking water that constitutes a ‘moderate’ risk (>10 colony-forming units (CFU)/100 mL of *E. coli* or thermotolerant coliforms per 100 mL) [1]. The UNICEF/World Health Organization Joint Monitoring Programme (JMP) differentiate as least hazardous ‘improved’ sources that are protected from contamination by nature of their design from ‘unimproved’ sources [2]. Among unimproved sources, direct consumption of surface waters is recognised as the most hazardous source type. In many instances, even water from improved sources may contain high levels of faecal indicator bacteria, particularly in rural areas [3]. Since logistical and resource issues such as transport and staff retention often prevent water quality testing [4,5], objective characterisation of contamination hazards at rural water sources is critical for management, so that unsafe sources can be prioritised and contamination risks reduced. 

One approach to identifying contamination risk is the systematic observation of hazards at or surrounding water sources, known as sanitary risk observation. This approach is often used in rural low- and middle-income country settings, where resources for training and equipment are limited. Examples of observation items used include proximity of water sources to pit latrines, lack of fencing around water sources to prevent livestock entry, or inadequate lining of well shafts. Where feasible, microbiological contamination of water can also be used to corroborate observed hazards alongside sanitary risk observation. Often faecal indicator bacteria (FIB) are used because of the challenges of implementing more sophisticated forms of microbiological testing in low-resource rural settings. Several such studies have found little or no relationship between observed sanitary risk and FIB [6,7,8], whilst others have found moderate but significant associations [9,10]. For some shallow groundwater sources, a significant relationship between contamination hazards and FIB has only been apparent during rainfall events [11].

Whilst sanitary risk observation is promoted as a means of managing the safety of community water supplies [12], robust and consistent observations of hazards under field conditions can be challenging. Despite promotion and use of sanitary risk observation for over two decades, consistency between individuals making hazard observations has only been studied very recently. In qualitative interviews, a panel of 26 water professionals reported difficulties in the consistent interpretation of sanitary risk inspection protocols [13]. In a study examining independent observations of the same water sources by different surveyors, we recently found only modest inter-observer agreement when recording sanitary risk [14], with the most experienced surveyor identifying more hazards than less experienced colleagues. However, it is unclear how inaccuracy in hazard observations through sanitary risk inspection affects subsequent analyses of microbiological contamination in relation to the identified hazards. Inaccurate hazard observation can be regarded as a form of exposure misclassification, the inaccurate measurement of risk [15]. Assuming that such misclassification is non-differential (i.e., it affects microbially contaminated and uncontaminated wells equally), it would lead to a tendency for the under-estimation or dilution of the strength of association between hazards and microbial contamination [16]. However, this tendency is moderated by factors such as prevalence of source contamination [16] and does not always hold true for individual studies [17].

Among faecal hazards relating to drinking-water contamination, systematic review evidence highlights the importance of livestock-related hazards, with 69% of studies identifying a significant relationship between animal husbandry and human diarrhoeal disease, increasing to 95% of studies examining pathogen-specific diarrhoea [18]. Given that many published studies have relied on reported livestock ownership rather than direct observations of contact between livestock and drinking-water, a recent systematic review [19] called for more robust methods for measuring livestock-related contact with drinking-water. Sanitary risk protocols include some observation items relating to animals or livestock (e.g., presence of animal faeces close to a wellhead), but it is unclear how consistently such observations can be made.

Building on earlier work [14], the objective of our study is therefore to assess how the identity of the surveyor conducting sanitary risk observations affects the strength of the association between observed hazards and microbiological contamination of drinking-water sources. As a secondary objective, we also aim to assess the importance of animal-related hazards for microbiological contamination, relative to other hazards, such as the structural integrity of water source protection measures or human faecal hazards.

## 2. Materials and Methods

### 2.1. Study Site, Sample Design and Recruitment

The study took place in ten villages in Siaya County, Kenya, an area near the shores of Lake Victoria where smallholder farming and livestock-keeping predominate and where households rely on a mix of rainwater harvesting, piped water, shallow wells, boreholes, and direct consumption of surface waters including those of Lake Victoria. A total of 234 households from these villages, participating in ongoing studies of livestock and human health [20], were randomly selected and recruited to the study. This sample was designed to detect a difference in microbial contamination of household stored water between cattle owners and those without cattle, so a balanced sample of 120 cattle-owning households and 114 households without cattle were recruited. 

All subjects gave their informed consent for inclusion before they participated in the study. The study was conducted in accordance with the Declaration of Helsinki, and the protocol was approved by the Ethics Committees of the Faculty of Social and Human Sciences, University of Southampton (reference: 31554; approval date: 12 February 2018) and the Kenya Medical Research Institute (reference: KEMRI/SERU/CGHR/091/3493, approval date: 17 October 2017).

### 2.2. Survey of Sanitary Risks at Water Sources

Drawing on published protocols [12] adapted through initial pilot fieldwork, sanitary risk inspection protocols were developed for the most prevalent source types in the study population, namely rainwater systems, protected and unprotected wells, springs, boreholes, and surface waters. Piped water sources were tested but excluded from sanitary risk inspection because of the logistical challenges of arranging inspections of supply infrastructure such as holding tanks, treatment units, and distribution pipelines. A team member experienced in sanitary risk observation (author JOO; Surveyor A) then recruited five other surveyors with varying levels of formal education and prior field survey experience. The less experienced and educated surveyors were recruited to be representative of those typically conducting sanitary risk inspection on rural water points. JOO then led an initial 4-day training and piloting events for this team. Results from the piloting events were evaluated and protocols adjusted before final application in the field.

A questionnaire survey was conducted with participant households, in which participants were asked to identify the source from which drinking-water stored in the home at the time of interview originated. Where water originated from a rainwater harvesting system that had since run dry, households identified an alternative drinking-water source. These household sources of drinking-water were then visited and a sanitary risk inspection conducted on each rainwater, spring, well, borehole or surface water extraction point used. No hazard inspection was conducted on piped water sources such as standpipes. In the first visit, all six surveyors independently visited each source and separately recorded any hazards observed, whilst, in the second visit, five surveyors independently visited each source after one surveyor dropped out. Because of logistical difficulties, there was sometimes a lag between source visits by different surveyors, particularly in the wet season. In the wet season, Surveyor A’s visits occurred a maximum of 16 days before his colleagues and 13 days after, with a median lag of 0 days and inter-quartile range of 5 days. In the dry season, the maximum lag was 14 days prior to his colleagues and 14 days after (median: 0 days; inter-quartile range: 2 days). We have previously reported inter-observer agreement in recording contamination hazards [14], but found no correlation between lag times between visits and difference in sanitary risk scores. Surveyors also recorded whether it had rained in the preceding week or days before each source visit. 

One surveyor (Surveyor B) additionally collected a water sample of approximately 500 mL from the source in a sterile polyethylene one litre bottle (Fisher Scientific, Loughborough, UK), testing water in situ for electro-conductivity, pH and turbidity using portable meters (COND3110 and Hanna Instruments HI 93703, respectively). Surveyor B also tested some piped samples in situ for free residual chlorine using SenSafe Water Check test strips, which are approved by the US Environmental Protection Agency (ITS Method 99-003) and for wells, measured depth to water table using a Solinst Model 102 M Coaxial Cable Water Level Meter. Samples were kept in a cooled container (4 °C) and transported within four hours to the Kenyan Medical Research Institute (KEMRI) laboratories in Kisian. Samples were either processed immediately or refrigerated at 4 °C and processed within 24 h. Sampling took place in wet and dry seasons between 10 April 2018 and 29 May 2018, and between 21 November 2018 and 20 February 2019.

### 2.3. Rainfall

In the absence of in situ gauge measures, rainfall data were derived from the Climate Hazard group InfraRed Precipitation with Station (CHIRPS) version 2 dataset [21]. CHIRPS is a quasi-global gridded rainfall product built on high-resolution satellite-based precipitation estimates combined with interpolated station data. It covers over 30 years’ rainfall estimates at high temporal (daily) and spatial (0.05 × 0.05 degree, approximately 5 × 5 km) resolutions, with particular value in areas where rain gauge density is sparse. Daily rainfall data for the fieldwork period were extracted from CHIRPS for each village.

### 2.4. Laboratory Microbiological Methods

The microbiological quality of drinking-water sources was assessed via faecal indicator bacteria (FIB), namely *E. coli* and intestinal enterococci. The presence of *E. coli* is associated with faecal contamination and it is the microorganism adopted by the World Health Organization (WHO, Geneva, Switzerland) (WHO, 2011) for verification of drinking-water microbial quality. The guideline value for *E. coli* is zero per 100 mL of water. Presence of intestinal enterococci also indicates faecal contamination, but these microorganisms may persist longer and be carried further than *E. coli* in the environment. Consequently, enterococci may indicate faecal contamination in water that might otherwise be missed. Although the WHO (WHO, 2011) has not established a guideline value for enterococci, it states that its detection should lead to consideration of further action. Furthermore, some studies suggest that gastrointestinal diseases are more strongly associated with the presence of enterococci than of *E. coli* [22]. Currently, the European Union’s Drinking Water Directive [23] includes intestinal enterococci as a parameter for audit monitoring with a standard of zero intestinal enterococci per 100 mL of water.

FIB enumeration was performed using membrane filtration according to International Standards Organization (ISO) standard methods (ISO 9308-1:2014 for *Escherichia coli* and total coliforms, and ISO 7899-2:2000 for intestinal enterococci). During initial pilot sampling, many 10 mL sample volumes and almost all 100 mL water sample volumes gave Too Numerous To Count (TNTC) results, so 0.1, 1 and 10 mL volumes per sample were filtered for the first visit for both FIB. Subsequent results from the first visit suggested many samples had less than 10 CFU/100 mL, so, in the second visit, four volumes (0.1, 1, 10 and 100 mL) were used. All samples were poured into a filtration unit containing approximately 10 mL of quarter-strength Ringer’s (QSR) solution and filtered through a 0.45 μm pore-size cellulose nitrate filter (Thermo Scientific, Waltham, MA, USA) using a vacuum pump (Fisher^®^). Filters for each volume were placed onto coliform chromogenic (CCE) agar (Difco^®^, Fisher Scientific, Loughborough, UK) in Ø 55 mm petri dishes (Fisher^®^). Plates were then incubated upside down for 24 ± 2 h at 37.0 ± 0.5 °C. Colonies coloured dark blue to violet were counted as *E. coli*, while pink to red-coloured colonies were recorded as presumptive (total) coliforms that were not *E. coli* [24]. Filters were placed onto Slanetz and Bartley agar (Oxoid^®^, Nepean, ON, Canada) in Ø 55 mm petri dishes (Fisher^®^) and incubated for 48 ± 2 h at 37.0 ± 0.5 °C. Raised colonies coloured red, maroon or pink were counted as presumptive intestinal enterococci [25]. Of the four volumes (0.1, 1, 10 and 100 mL) filtered per sample, the plate with the highest countable volume (100 mL) that was not TNTC was used for enumeration. All FIB results were expressed as colony-forming units (CFU) per 100 mL. All samples were processed in the laboratory without staff having knowledge of their origins.

### 2.5. Analysis of Sanitary Risk Scores versus Bacterial Contamination of Water Sources

To characterise contamination hazards from sanitary risk observations, an overall percentage sanitary risk score for each surveyor was calculated as the proportion of observable contamination hazards that were present at each score. As individual observation checklist items varied by source type, these items were classified into four domains (see Supplementary Table S2): items relating to faecal contamination by animals (e.g., footprints or animal faeces at a water point; lack of an intact fence or wall around a water point; branches where birds might rest overhanging roof catchments for rainwater harvesting or bird droppings on roof catchments); items relating to faecal contamination from humans (e.g., signs of open defecation; proximity of latrines); non-faecal contamination hazards (e.g., proximity of waste dumps; dirty buckets); and hazards that compromised source protection measures (e.g., lack of shaft lining, lack of an intact concrete apron or soakaway channel at a well; lack of a moveable inlet pipe to a rainwater harvesting tank). Separate percentage risk scores were calculated for these four hazard domains. 

To evaluate CHIRPS rainfall against field observations prior to model-fitting, the area under a receiver operating characteristic (AUC) curve was calculated for CHIRPS-derived rainfall in the previous week against rainfall occurrence in the previous week reported by the water sampling field team. The AUC was 0.90 (*n* = 191), suggesting good agreement between field observations and CHIRPS data, so CHIRPS-derived rainfall was subsequently examined in relation to FIB. 

Logistic regression modelling in Stata v16 [26] was then used to examine the relationship between sanitary survey observations and high contamination (>150 CFU/100 mL) of water points with faecal indicator bacteria. Logistic regression was used to avoid difficulties handling samples with left- or right-censored bacteria counts outside the limits of detection (<1 CFU/100 mL or Too Numerous To Count) [27]. The threshold value was chosen so that at least five samples were classified as highly contaminated and not highly contaminated per source type for both FIB, facilitating subsequent model fitting. Separate models were fitted for *E. coli* and intestinal enterococci, with robust regression to account for clustering of bacteria counts where two samples were taken from the same source. This approach was initially used to examine FIB in relation to total rainfall over periods of one day up to ten days preceding sampling, comparing models for each period using the Akaike Information Criterion (AIC). Rainfall for the period that best explained FIB was used in the subsequent modelling of FIB. Alongside the overall and four domain percentage sanitary risk scores, CHIRPS-derived rainfall in the seven days preceding sampling and source type (classed as rainwater, groundwater or surface water) were also included as explanatory variables. Following univariate model fitting, source type was included as a covariate in a set of bivariate regression models, alongside overall and domain sanitary risk scores. Since all surface water points lacked any structures to protect them from contamination, such sources were excluded from the model examining hazard scores for compromised source protection. Similarly, since no observations were made of human faecal contamination risks for rainwater systems, such sources were excluded from the bivariate model examining hazard scores for human faecal contamination. To examine the sensitivity of FIB predictive models to the identity of the sanitary survey staff member, separate logistic regression models were fitted in turn with sanitary risk records from the five surveyors participating in both survey visits. Finally, we also fitted a pooled logistic regression model predicting high FIB to the subset of water points visited by each of these five surveyors, testing for interactions between percentage sanitary risk score and surveyor identity. For shallow wells, we also calculated the Pearson’s correlation coefficient between logged FIB counts and depth to water table.

## 3. Results

### 3.1. Sampling of Water Sources

Since some households share the same water points, the water sampling surveyor visited 85 water points in the first fieldwork period and 143 in the second period, a total of 228 visits. However, water was unavailable for sampling at four sources in the first period and 40 sources in the second visit. Sources lacking water included broken pipes used by households, 17 rainwater systems, 13 taps, two boreholes, and six surface water sources. In the first period, there was no site access at a further three sources among the 184 where water was available, preventing sampling. This left 181 microbiological samples in total. One of these samples was lost during laboratory processing.

### 3.2. Microbiological Contamination of Water Sources

**Figure 1:**
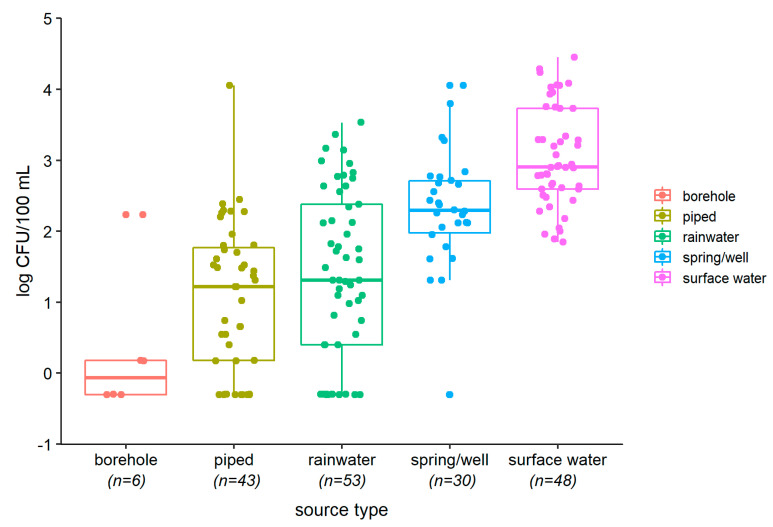
Boxplots showing levels (colony-forming units (CFU)/100 mL) of *E. coli* by source type (superimposed dots show bacteria counts for individual samples).

**Figure 2:**
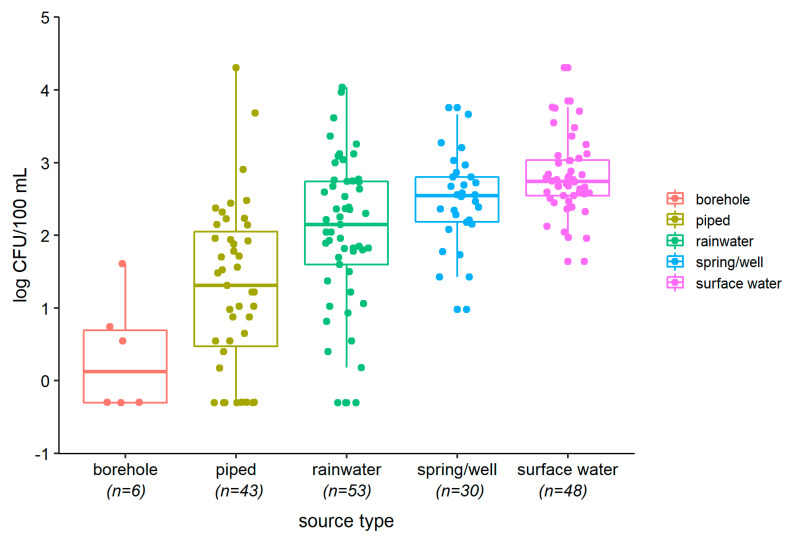
Boxplots showing levels (CFU/100 mL) of intestinal enterococci by source type (superimposed dots show bacteria counts for individual samples).

Figure 1 and Figure 2 show that, for both *E. coli* and intestinal enterococci, median contamination was greatest for surface waters, followed by wells and springs, rainwater, and then piped water. The small number of sampled boreholes had low contamination. Bacterial counts from rainwater showed the greatest variation. Surface waters were highly turbid, whilst groundwaters, particularly borehole water, had high electro-conductivity (Supplementary Table S1). Five of seven piped water samples tested had free residual chlorine below 0.2 mg/L, the recommended minimum level for preventing recontamination.

### 3.3. Sanitary Risk Observations and Rainfall Patterns

Table 1 shows mean percentage sanitary risk scores based on each surveyor’s observations and by source type. Surveyor A, the most experienced surveyor, recorded the most hazards overall and the most faecal hazards. However, Surveyor C recorded the most non-faecal hazards and Surveyor B the most instances of compromised protection measures (e.g., cracked concrete aprons for wells). Overall percentage hazard scores were greatest for surface water sources, followed by groundwater sources, and lowest for rainwater harvesting systems.

Mean rainfall was 4.1 and 34.3 mm in the day and week preceding sampling events, respectively. There was no rainfall the day before 78 (43%) sampling events and no rainfall in the week before 27 (15%) sampling events.

### 3.4. Hazards and Source Contamination

Table 2 shows unadjusted odds ratios for risk factors for high *E. coli* counts (>150 CFU/100 mL) in sampled water by source type, sanitary risk scores from the surveyor collecting samples (Surveyor B), and CHIRPS-derived rainfall in the week preceding sampling. Table 3 shows these odds ratios for high intestinal enterococci counts (>150 CFU/100 mL), again based on sanitary risk observations from the surveyor who collected water samples. Relative to piped water, samples from surface and groundwater sources but not rainwater had significantly higher odds of high contamination with *E. coli*. Odds of high contamination with intestinal enterococci were greater than piped water for groundwater, rainwater, and surface water sources. Rainfall in the week preceding sampling significantly increased the odds of water contamination with intestinal enterococci, but not *E. coli*. Whilst almost all hazard scores were significantly related to high bacterial contamination in univariate analysis, after adjusting for source type (and preceding rainfall for intestinal enterococci), only the animal-related hazard score remained significant for both faecal indicator bacteria groups (Table 2 and Table 3). This adjusted effect was modest: predicted probability of high contamination rose from 0.53 and 0.36 to 0.85 and 0.76, respectively, for enterococci and *E. coli* as the animal-related hazard score increased from zero to 100%.

For both *E. coli* and intestinal enterococci, adjusted coefficients for overall hazard scores remained insignificant when the other four surveyors’ scores were substituted for those of the surveyor conducting water sampling (Supplementary Table S3). The adjusted odds ratio for animal-related hazard scores was insignificant for three of the other four observers, despite being significant for observations by the surveyor collecting water samples. For Surveyor C, the adjusted odds ratio for *E. coli* was marginally significant for animal-related hazards, but not when unadjusted.

Table 4 shows the odds ratios for risk factors for high contamination with *E. coli* and intestinal enterococci for all 77 water points visited by every surveyor. There were no significant interactions between surveyor identity and percentage sanitary risk score, indicating no significant association between risk scores and high FIB contamination for any of the surveyors. In these multivariate models, only a surface water source type (e.g., dam or lake) was significantly associated with high FIB contamination.

**Figure 3:**
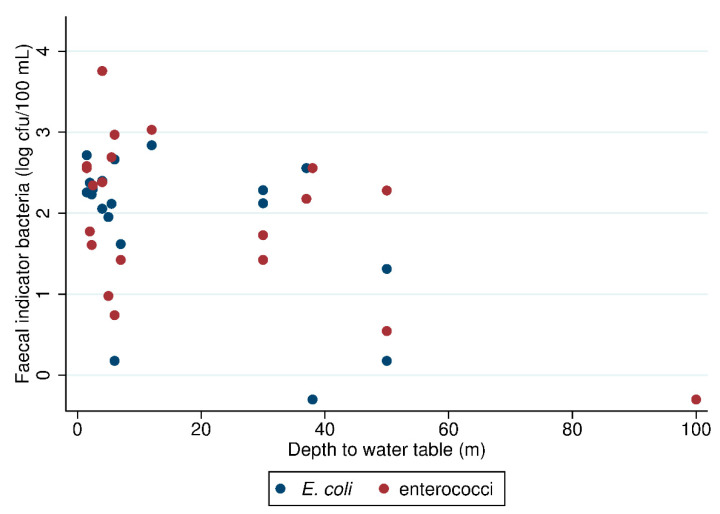
*E. coli* and intestinal enterococci counts versus depth to water table for 27 samples from 19 hand-dug wells.

As shown in Figure 3, for shallow wells, log *E. coli* and enterococci counts declined significantly with increasing depth to water table (R = −0.73; *p* < 0.001 and R = −0.65; *p* < 0.001, respectively).

## 4. Discussion

Our study provides some evidence that inter-observer variation in the recording of hazards could affect estimates of the relationship between hazards and FIB contamination. After controlling for source type, significant relationships were identified between animal-related hazards and FIB for Surveyors E and C, but not for three other surveyors. No such inter-observer variation in association with FIB contamination was identified for overall percentage risk scores, however (Table 4). Assuming the most experienced surveyor (A) more accurately recorded hazards, exposure misclassification simulation studies [16] suggest his hazard observations would tend to correlate more strongly with FIB than those of his colleagues. However, this tendency does not hold true for all studies [17] and assumes non-differential hazard misclassification (i.e., equal chances of hazard misclassification at contaminated and uncontaminated sources). Thus, the impact of hazard misclassification on strength of association with FIB is complex and unpredictable. Alongside other factors, the complex effects of hazard misclassification could thus in part account for the varying strength of association between FIB and sanitary risk scores reported in previous studies, e.g., [8,10]. 

We found only moderate inter-observer agreement in hazard recording at our Kenyan study site [14]. In contrast, in a previous study of inter-observer agreement concerning groundwater sources in urban Ghana [28], we found minimal disagreement. However, despite this Ghanaian study, qualitative interviews with water sector professionals [13] suggest that inconsistency and ambiguity in sanitary risk assessment is perceived as a widespread problem and therefore our findings should have wider applicability.

After adjusting for source type, there was limited evidence that higher animal-related hazard scores were associated with greater FIB levels. The adjusted and unadjusted relationship was significant for the surveyor collecting water samples for both intestinal enterococci and *E. coli*, for *E. coli* only for Surveyor C, but not for the three other surveyors. In general, more hazards were observed at the more bacteriologically contaminated source types such as surface waters, so overall scores and scores for other hazard types were no longer significant after controlling for source type. There was thus only limited evidence for a link between animal or livestock contact with water sources and FIB contamination in our study. This contrasts with evidence that sheep numbers increased the risk of *Cryptosporidium* spp. contamination of surface waters in India [29,30] and for animal faecal contamination of tubewells [19]. 

Aside from sanitary risk scores, depth to water table were significantly related to FIB contamination. Vertical separation between groundwater and surface hazards is known to reduce risks of microbial contamination through bacteria attenuation during water transport through the soil matrix [31], as identified in previous studies. A study of Bangladeshi tubewells found that water table depth predicted contamination, but the structural integrity of the well platform did not predict *E. coli* contamination [32]. In rural Kenyan wells, both water table depth and sanitary risk observations predicted such contamination [33]. Since we found significantly lower *E. coli* and intestinal enterococci counts in wells deeper than 40 m (Figure 3), this suggests that investment in depth probe equipment for field teams is justified as an objective means of characterizing shallow well contamination risk. 

After controlling for source type, we found a significant increase in intestinal enterococci, but not *E. coli*, following high rainfall in the preceding week (Table 3 and Table 4). The relationship with intestinal enterococci may reflect the flushing of contamination into water sources by rains and, in some shallow well systems, the rising of the water table following rainfall [11]. However, it is unclear why there was no similar increase in *E. coli* counts. High satellite-derived rainfall preceding sampling has previously been identified as a risk factor for thermotolerant coliform contamination of household stored water in Rwanda [34] and *E. coli* contamination of shallow wells in urban Kisumu, Kenya [35]. This suggests that there is potential to use satellite-rainfall to predict and model microbial contamination in national scale data sets, such as household surveys incorporating water quality modules.

The distribution of water quality parameters by source type provides some further insights into patterns of water source contamination and use. Notably, the presence of FIB in piped water has been reported by other studies in low and middle-income countries [3]. It reflects inadequate residual chlorine below the 0.2 mg/L recommended by WHO [36] at consumer endpoints. The study site population’s preference for rainwater, noted elsewhere in rural western Kenya [37], may reflect its low turbidity and also low electro-conductivity and saltiness of taste relative to borehole water.

Our findings are affected by several limitations. Whilst a contamination pathway such as a cracked concrete apron around a well may be present, it may not be active at the time of sampling and so not reflected in FIB counts. For example, repeated weekly testing of shallow wells for *E. coli* and intestinal enterococci in Thailand suggests that transient contamination peaks may be missed by cross-sectional sampling [38], with similar temporal variability in FIB from a study of shallow wells in Cambodia [39]. Our protocol involved tracing water sources used for drinking by participating households in different seasons. Whilst this meant that our sample reflected the diversity of source types that the population used, heterogeneity in hazards at different source types presented challenges for analysis. For example, to enable sufficient contaminated and uncontaminated samples for robust model-fitting across all source types, we had to adopt a threshold of 150 CFU/100 mL in regression analysis. This differs from the long-established practice of using 10 or 100 CFU/mL thresholds to define water contamination classes [40]. The survey team’s inability to observe some hazards could have affected our findings. In calculating overall and component percentage sanitary risk scores, we excluded hazard checklist items that could not be observed. FIB have been criticised as inadequate surrogates for assessing the presence of viral and protozoan pathogens in water sources [41], which may respond differently to FIB under stress from environmental factors. Rather than relying on FIB, similar future studies could enumerate pathogens specific to animal hosts of concern (e.g., *Cryptosporidium* spp.) or bacteriophages such as somatic coliphages. The latter have been considered by the US Environmental Protection Agency as possible viral indicators of faecal contamination for ambient water quality [42]. 

Repeated, longitudinal microbiological testing of a small number of rural water points, coupled with repeated sanitary risk observations, could provide stronger epidemiological evidence concerning the links between ephemeral contamination hazards and FIB. Studies examining the relationship between observed hazards at water points and microbial contamination have overwhelmingly tested for FIB (e.g., [6,7]). Rather than FIB, there would be merit in testing for viral indicators (bacteriophages) and specific pathogens (e.g., *Campylobacter* spp.) in relation to specific hazards and transmission pathways (e.g., proximity of poultry to water points). Since WHO has released revised sanitary risk protocols since we conducted our fieldwork [43], there would also be scope to repeat this study using these updated observation protocols. 

## 5. Conclusions

In this study, we examined how surveyor identity affected the strength of relationship between observed contamination hazards and FIB levels in water sources. We found that adjusted and unadjusted odds ratios for animal-related hazards were associated with greater risk of water contamination with both *E. coli* and intestinal enterococci. However, this was only true for hazard observations made by one surveyor amongst a team of five independently inspecting water sources. This suggests that the strength of association between hazards and microbiological contamination of water sources can be sensitive to inter-observer variation in hazard recording. Shallow well contamination with both FIB decreased with depth to water table, whilst rainfall in the week preceding sampling increased risk of high intestinal enterococci contamination. On the basis of these findings following calls made elsewhere [13], we recommend revision to existing sanitary risk protocols, so as to enable more consistent recording of hazards. In particular, our findings suggest that investment is justified in equipment for measuring contamination risks objectively, particularly depth probes for shallow wells. Our findings do not identify a particular sub-group of hazard observations that are correlated with high FIB. However, observers more consistently identified compromised source protection measures (e.g., cracked concrete aprons or missing/broken drainage channels at wellheads) than observations of hazards in the surrounding environment (e.g., signs of open defecation or uncollected solid waste). The latter thus require revision and greater surveyor training.

## Figures and Tables

**Table 1:** Mean sanitary risk scores for each surveyor and by source type (Surveyor A: most experienced surveyor; Surveyor B: surveyor collecting water samples; Surveyor D: participated in wet season fieldwork only. Surveyor A’s visits were a median 0 days apart from those of his colleagues, with an inter-quartile range of 2 days in the dry season and 5 days in the wet season).

	*n*	Animal Faecal Hazard Score	Human Faecal Hazard Score	Non-Faecal Hazard Score	Protection Measures Compromised-Score	Overall Risk Score
Surveyor						
Surveyor A	119	69.0%	46.1%	45.8%	29.4%	54.8%
Surveyor B	130	51.7%	39.4%	42.2%	58.0%	44.7%
Surveyor C	121	64.5%	43.0%	55.5%	21.8%	51.6%
Surveyor D	54	40.4%	29.5%	40.5%	16.9%	40.6%
Surveyor E	116	54.0%	27.9%	44.0%	18.8%	42.3%
Surveyor F	131	35.4%	26.3%	39.1%	16.9%	36.4%
Source type						
groundwater	192	77.4%	30.6%	32.8%	36.7%	40.8%
rainwater	225	36.2%	0.0%	33.2%	31.7%	32.7%
surface water	254	50.4%	71.7%	64.2%	100.0%	59.9%

**Table 2:** Odds ratios for risk factors for high contamination (>150 CFU/100 mL) of source water with *E*. *coli* based on logistic regression of 130 samples from 93 water points.

Risk Factor	Univariate Odds Ratio (Confidence Intervals)	*p* Value	Odds Ratio Adjusted for Source Type (Confidence Intervals)	*p* Value
Source type (reference: piped water)				
Groundwater	4.89 (1.84–13.01)	0.001		
Rainwater	1.73 (0.70–4.27)	0.236		
Surface water	30.63 (16.74–89.38)	<0.001		
Rainfall in 7 days preceding sampling (mm)	1.00 (0.99–1.01)	0.336		
Sanitary risk score Overall (%)	1.05 (1.03–1.08)	<0.001	1.01 (0.99 to 1.04)	0.363
Human faecal hazard (%)	1.01 (1.00–1.02)	0.007	1.00 (0.99–1.01) ^a^	0.915
Animal faecal hazard (%)	1.02 (1.01–1.03	<0.001	1.02 (1.00–1.03)	0.014
Non-faecal hazard (%)	1.02 (1.01–1.04)	0.006	1.01 (0.99–1.02)	0.521
Protection measures compromised (%)	1.03 (1.02–1.05)	<0.001	1.00 (0.98 to 1.03) ^b^	0.747

^a^ excludes rainwater systems; ^b^ excludes surface water points.

**Table 3:** Odds ratios for risk factors for high contamination (>150 CFU/100 mL) of source water with intestinal enterococci based on logistic regression of 130 samples from 93 water points.

Risk Factor	Univariate Odds Ratio (Confidence Intervals)	*p* Value	Multivariate Odds Ratio (Confidence Intervals)	*p* Value
Source type (reference: piped water)				
Groundwater	5.94 (2.00 to 17.63)	0.001		
Rainwater	3.64 (1.37 to 9.69)	0.010		
Surface water	32.49 (9.95 to 106.1)	<0.001		
Rainfall in 7 days preceding sampling (mm)	1.01 (1.00 to 1.02)	0.008		
Sanitary risk score Overall (%)	1.04 (1.01 to 1.06)	0.002	1.01 (0.98 to 1.04)	0.648
Human faecal hazards (%)	1.02 (1.01 to 1.03)	<0.001	1.00 (0.99 to 1.02) ^a^	0.830
Animal faecal hazard (%)	1.02 (1.00 to 1.03)	0.010	1.02 (1.00 to 1.03)	0.031
Non-faecal hazard (%)	1.01 (1.00 to 1.03)	0.080	1.00 (0.98 to 1.01)	0.697
Protection measures compromised (%)	1.03 (1.01 to 1.04)	<0.001	1.00 (0.97 to 1.04) ^b^	0.942

^a^ excludes rainwater systems; ^b^ excludes surface water points.

**Table 4:** Odds ratios for risk factors for high contamination (>150 CFU/100 mL) of source water with *E. coli* and intestinal enterococci based on multivariate logistic regression of 99 samples from 77 water points visited by all five surveyors (Surveyor D, who did not participate in dry season fieldwork, is excluded).

	*E. coli*		Intestinal Enterococci	
Risk Factor	Odds Ratio (Confidence Intervals)	*p* Value	Odds Ratio (Confidence Intervals)	*p* Value
Source type (reference: groundwater)				
Rainwater	0.38 (0.11 to 1.29)	0.122	0.85 (0.28 to 2.63)	0.779
Surface water	11.17 (3.01 to 41.42)	<0.001	6.82 (1.65 to 28.14)	0.008
Rainfall in 7 days preceding sampling (mm)			1.02 (1.00 to 1.04)	0.106
Sanitary risk score Overall (%)	0.97 (0.94 to 1.01)	0.081	0.99 (0.96 to 1.03)	0.625
Surveyor interaction with risk score (reference: Surveyor A)				
Surveyor B	1.02 (0.99 to 1.05)	0.122	1.01 (0.99 to 1.03)	0.470
Surveyor C	1.02 (0.99 to 1.04)	0.193	1.00 (0.98 to 1.03)	0.753
Surveyor E	1.01 (0.99 to 1.04)	0.429	1.00 (0.97 to 1.02)	0.854
Surveyor F	1.01 (0.99 to 1.04)	0.293	1.00 (0.98 to 1.03)	0.869

## Supplementary Materials

The following are available online at https://www.mdpi.com/1660-4601/17/24/9192/s1, Table S1: electro-conductivity and turbidity of water samples from different sources, Table S2: Classification of hazards observed at four rural water source types, Table S3: Odds ratios for hazards recorded by Surveyor A versus high contamination (>150 cfu/100 mL) of source water with *E. coli* based on logistic regression of 119 samples from 89 water points, Table S4: Odds ratios for hazards recorded by Observer Surveyor A versus high contamination (>150 cfu/100 mL) of source water with intestinal enterococci, based on logistic regression of 119 samples from 89 water points (a excludes rainwater systems; b excludes surface water points), Table S5: Odds ratios for hazards recorded by Surveyor C versus high contamination (>150 cfu/100 mL) of source water with *E. coli* based on logistic regression of 121 samples from 89 water points, Table S6: Odds ratios for hazards recorded by Surveyor C versus high contamination (>150 cfu/100 mL) of source water with intestinal enterococci, based on logistic regression of 121 samples from 89 water points, Table S7: Odds ratios for hazards recorded by Surveyor E versus high contamination (>150 cfu/100 mL) of source water with *E. coli* based on logistic regression of 116 samples from 87 water points, Table S8: Odds ratios for hazards recorded by Surveyor E versus high contamination (>150 cfu/100 mL) of source water with intestinal enterococci, based on logistic regression of 116 samples from 87 water points, Table S9: Odds ratios for hazards recorded by Surveyor F versus high contamination (>150 cfu/100 mL) of source water with *E. coli* based on logistic regression of 131 samples from 93 water points, Table S10: Odds ratios for hazards recorded by Surveyor F versus high contamination (>150 cfu/100 mL) of source water with intestinal enterococci, based on logistic regression of 131 samples from 93 water points.

## References

[1] Bain R., Cronk R., Hossain R., Bonjour S., Onda K., Wright J., Yang H., Slaymaker T., Hunter P., Pruss-Ustun A. (2014). Global assessment of exposure to faecal contamination through drinking water based on a systematic review. Trop. Med. Int. Health.

[2] WHO/UNICEF (2017). 2017 Annual Report—WHO/UNICEF Joint Monitoring Programme for Water Supply, Sanitation and Hygiene.

[3] Bain R., Cronk R., Wright J., Yang H., Slaymaker T., Bartram J. (2014). Fecal Contamination of Drinking-Water in Low- and Middle-Income Countries: A Systematic Review and Meta-Analysis. PLoS Med..

[4] Peletz R., Kisiangani J., Bonham M., Ronoh P., Delaire C., Kumpel E., Marks S., Khush R. (2018). Why do water quality monitoring programs succeed or fail? A qualitative comparative analysis of regulated testing systems in sub-Saharan Africa. Int. J. Hyg. Environ. Health.

[5] Wright J., Liu J., Bain R., Perez A., Crocker J., Bartram J., Gundry S. (2014). Water quality laboratories in Colombia: A GIS-based study of urban and rural accessibility. Sci. Total Environ..

[6] Ercumen A., Naser A.M., Arnold B.F., Unicomb L., Colford J.M., Luby S.P. (2017). Can Sanitary Inspection Surveys Predict Risk of Microbiological Contamination of Groundwater Sources? Evidence from Shallow Tubewells in Rural Bangladesh. Am. J. Trop. Med. Hyg..

[7] Luby S.P., Gupta S.K., Sheikh M.A., Johnston R.B., Ram P.K., Islam M.S. (2008). Tubewell water quality and predictors of contamination in three flood-prone areas in Bangladesh. J. Appl. Microbiol..

[8] Misati A.G., Ogendi G., Peletz R., Khush R., Kumpel E. (2017). Can Sanitary Surveys Replace Water Quality Testing? Evidence from Kisii, Kenya. Int. J. Environ. Res. Public Health.

[9] Wright J.A., Cronin A., Okotto-Okotto J., Yang H., Pedley S., Gundry S.W. (2013). A spatial analysis of pit latrine density and groundwater source contamination. Environ. Monit. Assess..

[10] Howard G., Pedley S., Barrett M., Nalubega M., Johal K. (2003). Risk factors contributing to microbiological contamination of shallow groundwater in Kampala, Uganda. Water Res..

[11] Godfrey S., Timo F., Smith M.D. (2006). Relationship between rainfall and microbiological contamination of shallow groundwater in Northern Mozambique. Water SA.

[12] World Health Organization (1997). Guidelines for Drinking-Water Quality. Volume 3: Surveillance and Control of Community Supplies.

[13] Pond K., King R., Herschan J., Malcolm R., McKeown R., Schmoll O. (2020). Improving Risk Assessments by Sanitary Inspection for Small Drinking-Water Supplies—Qualitative Evidence. Resources.

[14] Okotto Okotto J., Wanza P., Kwoba E., Yu W., Dzodzomenyo M., Thumbi S.M., Trajano Gomes da Silva D., Wright J. (2020). An assessment of inter-observer agreement of water source classification and sanitary risk observations. Expo. Health.

[15] Kirkwood B., Sterne A.C. (2003). Essential Medical Statistics.

[16] Jurek A.M., Greenland S., Maldonado G., Church T.R. (2005). Proper interpretation of non-differential misclassification effects: Expectations vs. observations. Int. J. Epidemiol..

[17] Sorahan T., Gilthorpe M.S. (1994). Non-differential misclassification of exposure always leads to an underestimate of risk: An incorrect conclusion. Occup. Environ. Med..

[18] Zambrano L.D., Levy K., Menezes N.P., Freeman M.C. (2014). Human diarrhea infections associated with domestic animal husbandry: A systematic review and meta-analysis. Trans. R. Soc. Trop. Med. Hyg..

[19] Penakalapati G., Swarthout J., Delahoy M.J., McAliley L., Wodnik B., Levy K., Freeman M.C. (2017). Exposure to Animal Feces and Human Health: A Systematic Review and Proposed Research Priorities. Environ. Sci. Technol..

[20] Thumbi S.M., Njenga M.K., Marsh T.L., Noh S., Otiang E., Munyua P., Ochieng L., Ogola E., Yoder J., Audi A. (2015). Linking Human Health and Livestock Health: A “One-Health” Platform for Integrated Analysis of Human Health, Livestock Health, and Economic Welfare in Livestock Dependent Communities. PLoS ONE.

[21] Funk C., Peterson P., Landsfeld M., Pedreros D., Verdin J., Shukla S., Husak G., Rowland J., Harrison L., Hoell A. (2015). The climate hazards infrared precipitation with stations—A new environmental record for monitoring extremes. Sci. Data.

[22] Barrell R.A., Hunter P.R. (2000). Microbiological standards for water and their relationship to health risk. Commun. Dis. Public Health.

[23] European Union (1998). Council Directive 98/83/EC on the Quality of Water Intended for Human Consumption.

[24] International Organization for Standardization (2014). Water Quality—Enumeration of Escherichia coli and Coliform Bacteria—Part 1: Membrane Filtration Method for Waters with Low Bacterial Background Flora.

[25] International Organization for Standardization (2000). Water Quality—Detection and Enumeration of Intestinal Enterococci—Part 2: Membrane Filtration Method.

[26] Statacorp LLC (2019). StataCorp Stata Statistical Software.

[27] McBride G. (2005). Using Statistical Methods for Water Quality Management: Issues, Problems, and Solutions.

[28] Yentumi W., dzodzomenyo M., Seshie-Doe K., Wright J. (2019). An assessment of the replicability of a standard and modified sanitary risk protocol for groundwater sources in Greater Accra. Environ. Monit. Assess..

[29] Daniels M.E., Shrivastava A., Smith W.A., Sahu P., Odagiri M., Misra P.R., Panigrahi P., Suar M., Clasen T., Jenkins M.W. (2015). Cryptosporidium and Giardia in Humans, Domestic Animals, and Village Water Sources in Rural India. Am. J. Trop. Med. Hyg..

[30] Daniels M.E., Smith W.A., Schmidt W.P., Clasen T., Jenkins M.W. (2016). Modeling Cryptosporidium and Giardia in Ground and Surface Water Sources in Rural India: Associations with Latrines, Livestock, Damaged Wells, and Rainfall Patterns. Environ. Sci. Technol..

[31] ARGOSS (2001). Guidelines for Assessing the Risk to Groundwater from Onsite Sanitation.

[32] Leber J., Rahman M.M., Ahmed K.M., Mailloux B., van Geen A. (2011). Contrasting Influence of Geology on E-coli and Arsenic in Aquifers of Bangladesh. Ground Water.

[33] Ferrer N., Folch A., Maso G., Sanchez S., Sanchez-Vila X. (2020). What are the main factors influencing the presence of faecal bacteria pollution in groundwater systems in developing countries?. J. Contam. Hydrol..

[34] Kirby M.A., Nagel C.L., Rosa G., Iyakaremye L., Zambrano L.D., Clasen T.F. (2016). Faecal contamination of household drinking water in Rwanda: A national cross-sectional study. Sci. Total Environ..

[35] Okotto-Okotto J., Okotto L.G., Price H.D., Pedley S., Wright J. (2015). A longitudinal study of long-term change in contamination hazards and shallow well quality in two neighbourhoods of Kisumu, Kenya. Int. J. Environ. Res. Public Health.

[36] World Health Organization (2011). Measuring Chlorine Levels in Water Supplies.

[37] Thomson P., Bradley D., Katilu A., Katuva A., Lanzoni M., Koehler J., Hope R. (2019). Rainfall and groundwater use in rural Kenya. Sci. Total Environ..

[38] Chuah C.J., Ziegler A.D. (2018). Temporal Variability of Faecal Contamination from On-Site Sanitation Systems in the Groundwater of Northern Thailand. Environ. Manag..

[39] Bennett H.B., Shantz A., Shin M., Sampson M.L., Meschke J.S. (2010). Characterisation of the water quality from open and rope-pump shallow wells in rural Cambodia. Water Sci. Technol. Water Supply.

[40] Lloyd B.J., Bartram J. (1991). Surveillance solutions to microbiological problems in water quality control in developing countries. Water Sci. Technol. Water Supply.

[41] Gleeson C., Gray N. (1997). The Coliform Index and Waterborne Disease: Problems of Microbial Drinking Water Assessment.

[42] US Environmental Protection Agency (2015). Review of Coliphages as Possible Indicators of Fecal Contamination for Ambient water Quality.

[43] World Health Organization (2020). Rainwater Collection and Storage.

