# Supplementary material for: Effect of Inter-Observer Variation on the Association between Contamination Hazards and the Microbiological Quality of Water Sources: A Longitudinal Study
Source: Int J Environ Res Public Health. 2020 Dec 9;17(24):9192. doi: 10.3390/ijerph17249192 (PMC7764753; doi:10.3390/ijerph17249192)
Supplement: Supplementary file 1 [file ijerph-17-09192-s001.zip › SourceWaterSuppFile1.pdf]

Okotto-Okotto et al: Effect of inter-observer variation on association between contamination hazards and microbiological quality of water sources: a longitudinal study

Table S1. electro-conductivity and turbidity of water samples from different sources.

Source type.	Mean turbidity – NTU (n*)	Mean electro-conductivity - µS (n*)
Piped or kiosk water	11.9 (37)	180.4 (38)
Borehole	12.0 (6)	1276.8 (6)
Well /spring	65.0 (27)	668.7 (28)
Rainwater	3.9 (52)	72.8 (53)
Surface water	352.0 (44)	384.8 (44)

*n= number of samples.
