# Supplementary material for: Effect of Inter-Observer Variation on the Association between Contamination Hazards and the Microbiological Quality of Water Sources: A Longitudinal Study
Source: Int J Environ Res Public Health. 2020 Dec 9;17(24):9192. doi: 10.3390/ijerph17249192 (PMC7764753; doi:10.3390/ijerph17249192)
Supplement: Supplementary file 1 [file ijerph-17-09192-s001.zip › SourceWaterSuppFile2.pdf]

Okotto-Okotto et al: Effect of inter-observer variation on association between contamination hazards and microbiological quality of water sources: a longitudinal study

**Hazard observation**	**Hazard type**
**Rainwater systems**	
Are there bird droppings on the roof catchment area?	Animal faecal hazard
Are there leaves / plants on the roof catchment area?	Other contamination hazard
Are there branches overhanging the roof catchment area?	Animal faecal hazard
Are there other contamination sources around the roof catchment area?	Other contamination hazard
Are the guttering channels that collect water dirty or filled with debris such as leaves?	Other contamination hazard
Is there a moveable inlet pipe from the gutter to the tank?	Protection compromised
Is there a filter box or a sieve at the tank inlet?	Protection compromised
Is there debris in the filter box?	Protection compromised
Is there any other point of entry to the tank that is not properly covered?	Protection compromised
Is there any defect in the walls or top of the tank (e.g. cracks) that could let water in?	Protection compromised
Is there a depression on top of the tank that would allow ponding?	Protection compromised
Is there a concrete floor under the tap or place where water is collected?	Protection compromised
Is the concrete floor cracked?	Protection compromised
Is the concrete floor dirty?	Other contamination hazard
Is the concrete floor broken?	Protection compromised
Is the bucket used to collect water left on the ground	Protection compromised
Does the bucket used to collect water look dirty?	Other contamination hazard

Is the water collection area inadequately drained?	Protection compromised
**Surface water**	
Is there human habitation visible upstream or uphill of where people collect water?	Other contamination hazard
Are any latrines visible upstream or uphill of where people collect water?	Human faecal hazard
Are there any places where garbage is lying upstream or uphill of where people collect water?	Other contamination hazard
Are there any cemeteries upstream or uphill of where people collect water?	Other contamination hazard
Are there any farm animals kept upstream or uphill of where people fetch water?	Animal faecal hazard
Are there signs of animals (e.g. footprints; faeces; animals present; feathers) within 3m of the water collection point?	Animal faecal hazard
Is there a wall or fencing to keep animals out?	Animal faecal hazard
Is there crop production upstream or uphill of where people fetch water?	Other contamination hazard
**Protected wells**	
Is there a latrine within 30m of the well?	Human faecal hazard
Is the nearest latrine on higher ground than the well?	Human faecal hazard
Is there any animal excreta in the vicinity of the well?	Animal faecal hazard
Is there any human excreta in the vicinity of the well?	Human faecal hazard
Is there any garbage in the vicinity of the well?	Other contamination hazard
Are there any cemeteries in the vicinity of the well?	Other contamination hazard
Are there any animal slaughter areas in the vicinity of the well?	Other contamination hazard
Is there any other contamination source in the vicinity of the well?	Other contamination hazard
Is the drainage poor, causing stagnant water on the cement floor?	Protection compromised
Is there a drainage channel for this well?	Protection compromised
Is the drainage channel broken, permitting ponding?	Protection compromised

Is there a wall or fencing to keep animals out?	Animal faecal hazard
Are there signs of animals within 3m of the water collection point?	Animal faecal hazard
Is there a concrete floor around the well?	Protection compromised
Is the concrete floor less than 1m wide?	Protection compromised
Is there any ponding on the concrete floor?	Protection compromised
Is the concrete cracked?	Protection compromised
Is the handpump loose at base?	Protection compromised
Was the cover of the well in place and does it fit?	Protection compromised
**Boreholes**	
Is there a latrine within 30m of the borehole?	Human faecal hazard
Is the nearest latrine on higher ground than the borehole?	Human faecal hazard
Is there any animal excreta in the vicinity of the borehole?	Animal faecal hazard
Is there any human excreta in the vicinity of the borehole?	Human faecal hazard
Is there any garbage in the vicinity of the borehole?	Other contamination hazard
Is there any cemeteries in the vicinity of the borehole?	Other contamination hazard
Is there any other contamination source in the vicinity of the borehole?	Other contamination hazard
Is the drainage poor, causing stagnant water on the cement floor?	Other contamination hazard
Is there a drainage channel for this well?	Protection compromised
Is the drainage channel broken, permitting ponding?	Protection compromised
Is there a wall or fencing to keep animals out?	Animal faecal hazard
Are there signs of animals within 3m of the water collection point?	Animal faecal hazard
Is there a concrete floor around the well?	Protection compromised
Is the concrete floor less than 1m wide?	Protection compromised

Is there any ponding on the concrete floor?	Protection compromised
Is the concrete cracked?	Protection compromised
Is the handpump loose at base?	Protection compromised

Supplemental table S2: Classification of hazards observed at four rural water source types
