# Supplementary material for: Effect of Inter-Observer Variation on the Association between Contamination Hazards and the Microbiological Quality of Water Sources: A Longitudinal Study
Source: Int J Environ Res Public Health. 2020 Dec 9;17(24):9192. doi: 10.3390/ijerph17249192 (PMC7764753; doi:10.3390/ijerph17249192)
Supplement: Supplementary file 1 [file ijerph-17-09192-s001.zip › SourceWaterSuppFile3.pdf]

Okotto-Okotto et al: Effect of inter-observer variation on association between contamination hazards and microbiological quality of water sources: a longitudinal study

Risk factor	Univariate odds ratio (confidence intervals)	P value	Odds ratio adjusted for source type (confidence intervals)	P value
Sanitary risk score Overall (%)	1.03 (1.01-1.06)	0.019	0.99 (0.96-1.03)	0.690
Human faecal hazard (%)	1.01 (1.00-1.02)	0.051	0.99 (0.97-1.01) [a]	0.319
Animal faecal hazard (%)	1.00 (0.99-1.01)	0.829	1.00 (0.99-1.02)	0.523
Non-faecal hazard (%)	1.03 (1.01-1.05)	0.005	1.01 (0.99-1.03)	0.568
Protection measures compromised (%)	1.03 (1.02-1.05)	<0.001	0.99 (0.96 to 1.01) [b]	0.320

Table S3: Odds ratios for hazards recorded by Surveyor A versus high contamination (>150cfu/100ml) of source water with *E. coli* based on logistic regression of 119 samples from 89 water points ([a] excludes rainwater systems; [b] excludes surface water points)

Risk factor	Univariate odds ratio (confidence intervals)	P value	Odds ratio adjusted for source type and rainfall (confidence intervals)	P value
Sanitary risk score Overall %	1.02 (1.00 to 1.06)	0.039	1.00 (0.97 to 1.04)	0.839
Human faecal hazards (%)	1.02 (1.01 to 1.03)	<0.001	1.00 (0.98 to 1.02) [a]	0.856
Animal faecal hazard (%)	0.99 (0.98 to 1.01)	0.470	1.00 (0.99 to 1.02)	0.797
Non-faecal hazard (%)	1.02 (1.01 to 1.04)	0.007	1.01 (0.99 to 1.03)	0.244
Protection measures compromised (%)	1.02 (1.01 to 1.04)	0.002	0.99 (0.97 to 1.02) [b]	0.675

Table S4: Odds ratios for hazards recorded by Surveyor A versus high contamination (>150cfu/100ml) of source water with intestinal enterococci, based on logistic regression of 119 samples from 89 water points ([a] excludes rainwater systems; [b] excludes surface water points)

Risk factor	Univariate odds ratio (confidence intervals)	P value	Odds ratio adjusted for source type (confidence intervals)	P value
Sanitary risk score Overall (%)	1.06 (1.03-1.08)	<0.001	1.01 (0.98-1.04)	0.431
Human faecal hazard (%)	1.01 (0.99-1.02)	0.290	0.99 (0.98-1.00) [a]	0.188
Animal faecal hazard (%)	1.01 (0.99-1.02)	0.393	1.02 (1.00-1.04)	0.031
Non-faecal hazard (%)	1.04 (1.02-1.06)	<0.001	1.02 (1.00-1.04)	0.068
Protection measures compromised (%)	1.03 (1.02-1.05)	<0.001	1.00 (0.97 to 1.03) [b]	0.951

Table S5: Odds ratios for hazards recorded by Surveyor C versus high contamination (>150cfu/100ml) of source water with *E. coli* based on logistic regression of 121 samples from 89 water points ([a] excludes rainwater systems; [b] excludes surface water points)

Risk factor	Univariate odds ratio (confidence intervals)	P value	Odds ratio adjusted for source type and rainfall (confidence intervals)	P value
Sanitary risk score Overall %	1.04 (1.01 to 1.07)	0.003	1.01 (0.98 to 1.04)	0.424
Human faecal hazards (%)	1.01 (1.01 to 1.02)	0.001	1.00 (0.98 to 1.01) [a]	0.760
Animal faecal hazard (%)	1.00 (0.99 to 1.02)	0.718	1.01 (1.00 to 1.03)	0.186
Non-faecal hazard (%)	1.03 (1.01 to 1.05)	0.011	1.01 (0.99 to 1.03)	0.325
Protection measures compromised (%)	1.02 (1.01 to 1.03)	0.001	1.00 (0.97 to 1.02) [b]	0.710

Table S6: Odds ratios for hazards recorded by Surveyor C versus high contamination (>150cfu/100ml) of source water with intestinal enterococci, based on logistic regression of 121 samples from 89 water points ([a] excludes rainwater systems; [b] excludes surface water points)

Risk factor	Univariate odds ratio (confidence intervals)	P value	Odds ratio adjusted for source type (confidence intervals)	P value
Sanitary risk score Overall (%)	1.05 (1.02-1.08)	0.002	1.00 (0.97-1.04)	0.922
Human faecal hazard (%)	1.00 (0.99-1.01)	0.490	0.99 (0.98-1.01) [a]	0.916
Animal faecal hazard (%)	1.00 (0.99-1.02)	0.609	1.00 (0.99-1.02)	0.640
Non-faecal hazard (%)	1.02 (1.01-1.04)	0.004	1.01 (1.00-1.03)	0.058
Protection measures compromised (%)	1.03 (1.02-1.04)	<0.001	0.99 (0.97 to 1.02) [b]	0.672

Table S7: Odds ratios for hazards recorded by Surveyor E versus high contamination (>150cfu/100ml) of source water with *E. coli* based on logistic regression of 116 samples from 87 water points ([a] excludes rainwater systems; [b] excludes surface water points)

Risk factor	Univariate odds ratio (confidence intervals)	P value	Odds ratio adjusted for source type and rainfall (confidence intervals)	P value
Sanitary risk score Overall %	1.04 (1.01 to 1.07)	0.009	1.02 (0.98 to 1.05)	0.359
Human faecal hazards (%)	1.01 (1.00 to 1.02)	0.020	1.00 (0.99 to 1.02) [a]	0.801
Animal faecal hazard (%)	1.00 (0.99 to 1.01)	0.988	1.01 (0.99 to 1.02)	0.369
Non-faecal hazard (%)	1.01 (1.00 to 1.03)	0.103	1.01 (0.99 to 1.03)	0.262

Protection measures compromised (%)	1.02 (1.01 to 1.03)	0.001	0.99 (0.96 to 1.02) [b]	0.477

Table S8: Odds ratios for hazards recorded by Surveyor E versus high contamination (>150cfu/100ml) of source water with intestinal enterococci, based on logistic regression of 116 samples from 87 water points ([a] excludes rainwater systems; [b] excludes surface water points)

Risk factor	Univariate odds ratio (confidence intervals)	P value	Odds ratio adjusted for source type (confidence intervals)	P value
Sanitary risk score Overall (%)	1.05 (1.02-1.07)	<0.001	0.97 (0.93-1.01)	0.115
Human faecal hazard (%)	1.00 (0.99-1.01)	0.592	0.98 (0.96-1.00) [a]	0.097
Animal faecal hazard (%)	1.01 (1.00-1.02)	0.106	1.00 (0.98-1.03)	0.731
Non-faecal hazard (%)	1.04 (1.02-1.05)	<0.001	1.01 (0.99-1.03)	0.319
Protection measures compromised (%)	1.03 (1.02-1.04)	<0.001	0.98 (0.95 to 1.02) [b]	0.433

Table S9: Odds ratios for hazards recorded by Surveyor F versus high contamination (>150cfu/100ml) of source water with *E. coli* based on logistic regression of 131 samples from 93 water points ([a] excludes rainwater systems; [b] excludes surface water points)

Risk factor	Univariate odds ratio (confidence intervals)	P value	Odds ratio adjusted for source type and rainfall (confidence intervals)	P value
Sanitary risk score Overall %	1.04 (1.01 to 1.06)	0.002	0.99 (0.95 to 1.03)	0.574
Human faecal hazards (%)	1.01 (1.00 to 1.02)	0.086	0.99 (0.97 to 1.01) [a]	0.169
Animal faecal hazard (%)	1.00 (0.99 to 1.02)	0.522	1.01 (0.98 to 1.03)	0.658
Non-faecal hazard (%)	1.03 (1.01 to 1.04)	0.001	1.01 (0.99 to 1.03)	0.531
Protection measures compromised (%)	1.02 (1.01 to 1.04)	<0.001	0.99 (0.95 to 1.04) [b]	0.751

Table S10: Odds ratios for hazards recorded by Surveyor F versus high contamination (>150cfu/100ml) of source water with intestinal enterococci, based on logistic regression of 131 samples from 93 water points ([a] excludes rainwater systems; [b] excludes surface water points)
